# A reservoir at the gates: nonhuman mammalian hosts for human schistosomiasis in western Africa and the critical challenge for elimination

**DOI:** 10.1186/s40249-025-01394-6

**Published:** 2026-01-12

**Authors:** Benjamin Sanogo, Isaac Echoru, Caitlin Jones, Caitlin Butala, Datao Lin, Hamma Maiga, Hugo Sámano-Sánchez, Moussa Sacko, Kokouvi Kassegne, Susan Christina Welburn

**Affiliations:** 1 Zhejiang University-University of Edinburgh International Institute, Zhejiang University, Haining, 314400 China; 2 Infection Medicine, Edinburgh Medical School, College of Medicine & Veterinary Medicine, The University of Edinburgh, Edinburgh, Scotland, EH8 9JZ UK; 3 Department of Parasitology, Zhongshan School of Medicine, Sun Yat-Sen University, Guangzhou, 510080 China; 4 Département de Laboratoire, Institut National de Santé Publique (INSP), Bamako, 1771 Mali; 5 School of Global Health, Chinese Center for Tropical Disease Research, Shanghai Jiao Tong University School of Medicine, Shanghai, 200025 China

**Keywords:** Schistosomiasis, Elimination, Zoonotic transmission, Nonhuman mammalian reservoir, Hybrid schistosomes, West Africa, Integrated disease control

## Abstract

**Background:**

Schistosomiasis, a snail-borne parasitic disease of public health and veterinary importance in tropical areas, is highly prevalent in sub-Saharan Africa, particularly in West Africa. The World Health Organization (WHO) has established ambitious goals of eliminating schistosomiasis as public health problem or interrupting its transmission by 2030. The zoonotic transmission of schistosomiasis involving nonhuman mammals (NHMs) complicates disease endemicity and hinders the attainment of these objectives. This study synthesized recent trends and the prevalence of human-infective schistosomes (HISs), including *Schistosoma mansoni*, *S. haematobium*, *S. haematobium* × *S. bovis* hybrids, *S. guineensis*, and *S. intercalatum*—across 16 West African countries.

**Methods:**

We conducted a systematic literature search from March 25 to April 30, 2025, across PubMed, Scopus, Embase, and Web of Science, to identify studies on HISs (*S. mansoni*, *S. haematobium*, *S. haematobium* × *S. bovis*, *S. guineensis*, and *S. intercalatum*) in NHMs in western Africa. In addition, we manually searched African Journal Online (AJOL) and screened the references of the included articles. The data were organized in Microsoft Excel 2021 and analyzed via GraphPad Prism to identify publication trends, NHM infection incidence, and species-specific positivity rates (with 95% *CIs*). The spatial distribution of HIS-infected NHMs was visualized with QGIS to pinpoint high-risk areas.

**Results:**

Four countries (Benin, Ghana, Nigeria, and Senegal) reported cases of HIS infection in NHMs with an overall prevalence of 8% (95% *CI*: 7–9%). Benin had the highest proportion of infected hosts (50%, 95% *CI*: 40–60%) and Senegal had the lowest proportion (5%, 95% *CI*: 4–6%). *Bos taurus* (60% prevalence) was the most affected species and served as a reservoir for *S. haematobium* × *S. bovis* hybrids, whereas *S. mansoni* exhibited an extensive distribution among rodent and primate hosts.

**Conclusion:**

For effective elimination, integrated control strategies—spanning NHM surveillance, snail intermediate host monitoring, and human mass drug administration—must be prioritized. Policy reforms should address zoonotic transmission risks, particularly in high-prevalence zones, to align interventions with the complex ecology of schistosomiasis in West Africa.

**Graphical abstract:**

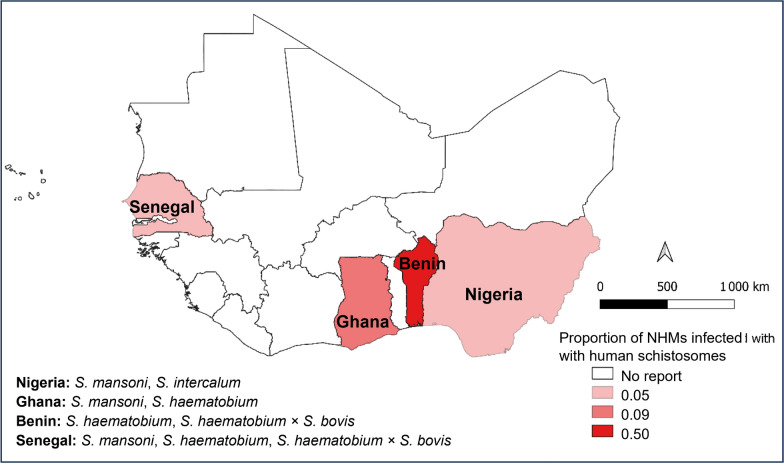

## Background

Schistosomiasis, a neglected tropical disease (NTD) of profound public health significance, remains a leading cause of morbidity in sub-Saharan Africa (SSA), particularly in West Africa, where more than 90% of the global burden resides [1–5]. In 2019, schistosomiasis accounted for 1.6 million disability-adjusted life years (DALYs), with a moderate prevalence (10–49%) across most West African nations [3, 6, 7]. While primarily transmitted through freshwater snails [8], the persistence of the disease is increasingly linked to zoonotic spillover from nonhuman mammalian (NHM) reservoirs, a critical yet underaddressed challenge for elimination efforts [9–11].

Human schistosomiasis in SSA is driven by *Schistosoma haematobium* (urogenital) and *S. mansoni* (intestinal), with sporadic cases of *S. intercalatum* and *S. guineensis* [4]. Chronic infection leads to fibrosis, anaemia, and organ damage, with *S. haematobium* implicated in bladder cancer and *S. mansoni* in hepatic complications [12–15]. High-risk groups—children, farmers, and women with water-dependent livelihoods—face disproportionate exposure due to socioeconomic and ecological factors [14, 16].

Current strategies, anchored in the mass drug administration (MDA) of praziquantel to school-aged children, fall short of the World Health Organization (WHO)’s 2030 goals for elimination (≤ 1% high-intensity infections) and transmission interruption [17–19]. The WHO’s “One Health” framework underscores the need to integrate human, animal, and environmental interventions, yet control programs rarely account for NHM reservoirs or emerging hybrid strains (e.g., *S. haematobium* × *S. bovis*), which complicate transmission dynamics [20–23].

The emergence of zoonotic hybrid schistosomes is propelled by anthropogenic and environmental changes that dismantle geographical barriers. Economic development, agricultural expansion, and climate change alter freshwater ecosystems, whereas human migration and global trade increase host species interactions [24]. Hybridization is a biological phenomenon that involves the meeting and interbreeding of two genetically distinct entities classified as separate species [25]. Specifically, shared water sources intensify contact between humans, livestock (e.g., *Bos taurus*), and wildlife (e.g., rodents), creating interfaces for schistosome spillover and hybridization [22, 26–28]. For example, *S. mansoni*—a major cause of human intestinal schistosomiasis—infects nonhuman primates in Kenya, Ethiopia, and Uganda [29, 30], whereas livestock and rodents in Senegal, Benin, and Ghana are confirmed reservoirs for *S. mansoni* and *S. haematobium*, with the prevalence reaching 50% in hotspots [31]. Hybridization between human- and animal-infective species (e.g., *S. haematobium* × *S. bovis*) may increase parasite adaptability, potentially undermining diagnostic and control efforts [23, 32]. However, surveillance systems and policies remain anthropocentric, neglecting NHM infections in burden estimates [22, 29].

### Context

Western Africa, comprising 16 mainland countries, is Africa's second most populous region (~ 467 million people) [33] and bears the highest schistosomiasis burden in SSA, with 85.4 million people requiring preventive chemotherapy [20]. *S. mansoni* and *S. haematobium* are endemic across the region [40], whereas zoonotic *S. haematobium* × *S. bovis* hybrids—reported in Mali, Senegal, Niger, and Benin—complicate transmission dynamics and control efforts [34, 35]. This high-transmission setting, combined with frequent human-animal-water contact, creates ideal conditions for persistent zoonotic transmission.

While human infective schistosomes are prevalent, the roles of NHM reservoirs and emerging hybrid schistosomes in perpetuating human schistosomiasis transmission across western Africa are unclear. In addition, how can this evidence reframe elimination strategies?

This systematic review aimed to synthesize evidence on human-infective schistosomes (HISs) in NHMs across 16 West African countries, highlighting prevalence trends, reservoir roles, and implications for elimination. By mapping zoonotic transmission risks and hybrid strains, we aim to inform integrated control strategies—spanning NHM surveillance, snail control, and community-based MDA—to close critical gaps in regional schistosomiasis control.

## Methods

### Search strategy

A systematic literature search was conducted from March 25 to April 30, 2025, across PubMed, Scopus, Embase, and Web of Science, to identify studies on HISs (*S. mansoni*, *S. haematobium*, *S. haematobium* × *S. bovis*, *S. guineensis*, and *S. intercalatum*) in NHMs in western Africa. The key search terms included the following: (i) pathogen-specific terms (*"*human schistosom"*, *"Schistosoma mansoni"*, "*Schistosoma haematobium*", “*Schistosoma intercalatum*”, “*Schistosoma guineensis*”, "intestinal bilharz*", "urogenital schistosom*", and "urogenital bilharz*"; (ii) transmission-related terms (*infection, prevalence, and epidemiology); and (iii) geographic terms (West Africa*, western African countries, Côte d'Ivoire, Mali, Niger, Nigeria, Guinea, Senegal, Gambia, Sierra Leone, Benin, Liberia, Togo, Ghana, Guinea-Bissau, Cabo Verde, and Mauritania), combined with Boolean operators (AND/OR). French search terms were included to capture regional literature. To ensure comprehensiveness, we manually searched African Journal Online (AJOL) and screened the references of the included articles. No restrictions were applied to publication dates, but only English/French studies were included.

The research protocol was registered on the International Platform of Registered Systematic Review and Meta-analysis Protocols (INPLASY) with the number INPLASY202570027 (10.37766/inplasy2025.7.0027).

### Concept

HISs—defined here as *S. mansoni*, *S. haematobium*, *S. haematobium* × *S. bovis* hybrids, *S. guineensis*, and *S. intercalatum*—are known to cause human schistosomiasis in endemic regions. NHM hosts include all wild and domestic mammals (e.g., rodents, livestock, primates) that may serve as reservoirs for HIS transmission, complicating control efforts through zoonotic spillover and hybridization. This framework underscores the need for integrated surveillance of HIS in both human and NHM populations to achieve elimination targets.

### Eligibility criteria

Studies were included if they (i) reported infections by HISs: *S. mansoni, S. haematobium, S. haematobium* × *S. bovis* hybrids, *S. guineensis*, or *S. intercalatum* in NHM hosts (wildlife or domestic animals) within any of the 16 West African countries; (ii) provided primary data on prevalence, distribution, or zoonotic implications; and (iii) were published in peer-reviewed journals or reputable gray literature without date restrictions to capture historical and contemporary trends. Excluded studies focused exclusively on nonmammalian hosts or experimental infections without field relevance.

### Study/source of evidence selection

The identified studies were imported into EndNote 21 (Clarivate Plc, London, United Kingdom) for deduplication, followed by a two-stage screening process. First, titles/abstracts were screened for reports of HISs (*S. mansoni*, *S. haematobium*, *S. haematobium* × *S. bovis*, *S. guineensis*, or *S. intercalatum*) in NHMs within western Africa. Eligible studies progressed to full-text review if they met the following three criteria: (i) documented natural (nonexperimental) infections; (ii) used validated diagnostic methods (parasitological, necropsy, immunological, or molecular techniques); and (iii) provided species-specific data. The reasons for exclusion were systematically recorded; no restrictions were applied to sample sizes to maximize evidence capture. Interrater reliability was assessed during screening, with discrepancies resolved by consensus.

### Extraction and analysis of data

Key data, including authorship, publication year, study location (country), design (cross-sectional/longitudinal), host species, sample size, diagnostic methods (e.g., parasitological, molecular), and infection outcomes (e.g., positive cases), were systematically extracted from the eligible studies. Studies encompassing multiple hosts (NHMs, humans, or snails) were noted. The data were organized in Microsoft Excel 2021 (Microsoft, Washington, USA) and analysed to determine (i) temporal/geographic publication trends in GraphPad Prism 10.6.1 (Dotmatics, Boston, USA), (ii) NHM infection incidence (with 95% *CIs*), and (iii) species-specific positivity rates. The spatial distribution of HIS-infected NHMs was visualized via QGIS 3.38.3 (QGIS Association, Grüt, Switzerland) to identify high-risk zones.

## Results

### Search results

**Fig. 1:**
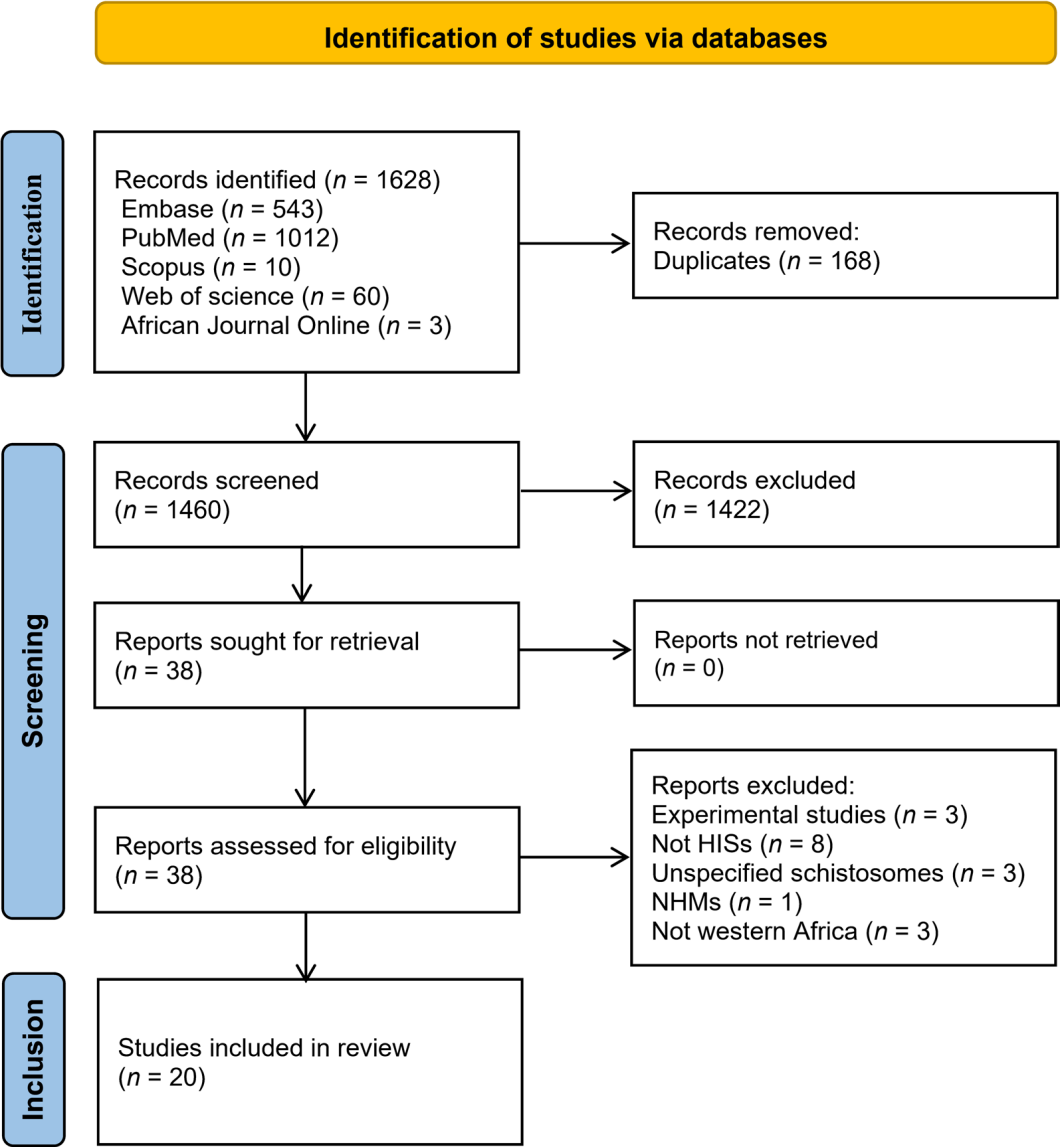
PRISMA flow diagram: literature search and study inclusion process for zoonotic schistosomiasis reservoirs in western Africa

Our systematic search identified 1628 studies, with 1460 remaining after duplicate removal. Title/abstract screening excluded 1422 articles because of the following: (i) experimental design, (ii) nonhuman-infective schistosomes, (iii) unspecified species, (iv) non-NHM hosts, or (v) nonwestern African focus. Among the 38 articles that progressed to full-text review, 20 met all the eligibility criteria for data extraction (Fig. 1). Notably, no relevant reviews or opinion pieces addressing HIS in western African NHMs were identified, highlighting a critical evidence gap in the zoonotic schistosomiasis literature.

### Characteristics of the included studies

**Fig. 2:**
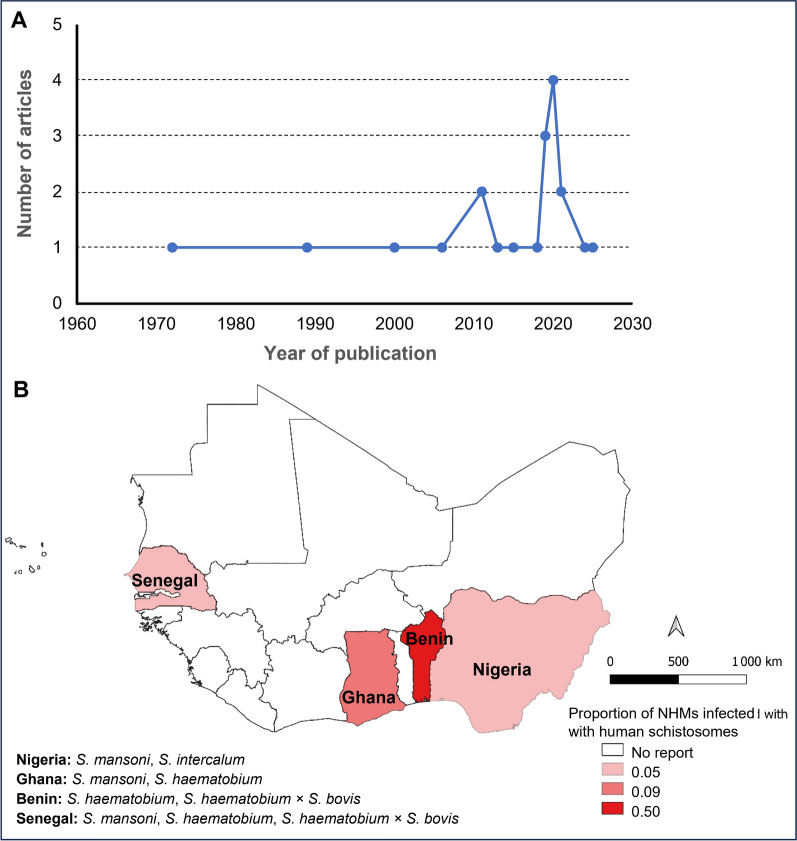
Research and epidemiological trends in zoonotic schistosomiasis in western Africa (1972–2025). **A** Temporal publication patterns. **B** Regional prevalence in animal reservoirs. *NHM* Nonhuman mammal (Source of map: generated with QGIS 3.38.3)

The publication frequency showed an irregular temporal distribution (Fig. 2A), with only three studies appearing between 1972 and 2000. Research output increased notably from 2006 onwards, peaking in 2019 during the active 2011–2021 period, although no publications were identified for 2022–2023. All 20 eligible studies represented original research, abstracts, or case reports from four West African nations, Senegal (*n* = 8), Nigeria (*n* = 5), Ghana (*n* = 4), and Benin (*n* = 3), revealing significant geographic concentration of zoonotic schistosomiasis research in the region.

### Sampling strategies and study characteristics

The included studies employed heterogeneous sampling approaches: convenience sampling predominated (13/20), followed by mixed methods [purposive/convenience: 2; simple random sampling: 2; random/convenience: 1; and singular purposive/random sampling (1 each)]. Sample sizes averaged 237 animals (range: 23–2299) across 29 NHM species (Table 1).

**Table 1:** Nonhuman mammalian reservoirs of human-infective schistosomes in western Africa: evidence from field studies (1972–2025)

Indicator	All	Benin	Ghana	Nigeria	Senegal
Number of NHMs sampled *n* (mean; SD)	4264 (237; 525)	260 (87; 65)	364 (92; 93)	371 (74; 71)	3,854 (479; 812)
Coverage of NHMs that host HISs for which data were available, *n* (*P* % at 95% confidence level)	358 (8%, 95% *CI*: 8–9%)	130 (50%, 95% *CI*: 40–60%)	33 (9%, 95% *CI*: 7–11%)	17 (5%, 95% *CI*: 3–7%)	198 (5%, 95% *CI*: 4–6%)
Number of areas/sites for which data on NHMs that host HISs were available	41	7	4	5	25
Number of datasets on NHMs that host IHSs for which data were available	27	5	4	5	11

*SD* standard deviation, *P* prevalence of infected NHMs, 95% *CI* confidence interval at the 95% confidence level, *NHM* Nonhuman mammal, *HIS* Human-infective schistosome

All studies were cross-sectional surveys or case investigations, primarily focusing on NHMs alone (14/20), although four studies assessed multiple hosts (NHMs/humans/snails [31, 36], NHMs/humans [37–39], or NHMs/snails [40] (Table 2). Wildlife constituted most surveyed populations (15/20), with free-ranging animals studied most frequently (13/15); livestock (farmed/abattoir-sampled [38, 41] and pet dogs were less represented. Only one study compared captive/free-roaming wildlife [42], whereas another integrated wildlife rodents/livestock [36]. The predominance of convenience sampling and wildlife-focused designs may reflect field challenges but underscores the need for standardized, multihost surveillance.

**Table 2:** Epidemiological characteristics of human-infective schistosome transmission in nonhuman mammalian hosts: western Africa, 1972–2023

Number	Authors and year of publication	Country of study	Survey type (target)	Sampling strategy	Nonhuman mammalian surveyed (*n* = sample size)	Biological material	Identification method	Positive cases/number of NHM surveyed (%)	Nonhuman mammalian species infected with human infective schistosomes	Human infective schistosome species	Reference
1	Abogye et al., 2019	Ghana	Cross-sectional (NHM)	Convenience sampling	Wildlife small and ruminant mammals: (Muridae, *n* = 41 and Bovidae, *n* = 159)	Postmortem feces collection	Microscopy (flotation)	4/200 (2.0%)	Muridae (*Thryonomys swinderianu*s)	*S. mansoni,* *S. haematobium*	[43]
2	Catalano et al., 2020	Senegal	Cross-sectional (NHM, humans and snails)	Convenience sampling	Wildlife small mammals: (Muridae, *n* = 237 and Soricidae, *n* = 14)	Postmortem worms and eggs’ (liver and intestines) collection	Necropsy and PCR	16/251 (6.4%)	Muridae (*Mastomys huberti*)	*S. mansoni*	[31]
3	Catalano et al., 2018	Senegal	Cross-sectional (NHM)	Convenience sampling	Wildlife small mammals: (Muridae,* n* = 393 and Soricidae, *n* = 27)	Postmortem worms and eggs’ (liver and intestines) collection	Necropsy and PCR	13/420 (3.1%)	Muridae (*Arvicanthis niloticus, n* = 1 and *M. huberti*,* n* = 7)	*S. mansoni, S. haematobium* × *S. bovis*	[44]
4	Catalano et al., 2019	Senegal	Cross-sectional (NHM)	Convenience sampling	Wildlife small mammals: (Muridae, *n* = 89)	Postmortem worms and feces collection	Necropsy, microscopy (mini-flotac), and PCR	21/89 (23.6%)	Muridae (*M. huberti*)	*S. mansoni and S. haematobium* × *S. bovis*	[45]
5	Duplantier et al., 2000	Senegal	Repetitive cross-sectional (NHM)	Convenience sampling	Wildlife small mammals: (Muridae, *n* = 2279; Sciuridae, *n* = 1; and Soricidae, *n* = 19)	Postmortem worms and eggs’ (liver and intestines) collection	Necropsy and microscopy (technique not specified)	116/2299 (5.0%)	Muridae (*A.* and *M. huberti)*	*S. mansoni*	[46]
6	Futagbi et al., 2015	Ghana	Cross-sectional (NHM)	Convenience sampling	Livestock (goats): small ruminant mammals (Bovidae, *n* = 35)	Postmortem feces collection	Necropsy and microscopy (Kato-Katz)	15/35 (42.9%)	Bovidae (Goats: *Capra aegagrus hircus*)	*S. haematobium*	[41]
7	Howells et al., 2011	Senegal	Cross-sectional (NHM)	Purposive and convenience sampling	Wildlife nonhuman primate mammals: (Hominidae, *n* = 132 and Cercopithecidae, *n* = 17)	Living animal’s feces	Microscopy (flotation and sedimentation)	4/149^*^ (2.7%)	Cercopithecidae (*Papio hamadryas papio*)	*S. mansoni*	[47]
8	Kincaid-Smith et al.,2024	Senegal	Cross-sectional (NHM and snails)	Convenience sampling	Wildlife small mammals (Muridae, *n* = 495; Soricidae, *n* = 39)	Postmortem worms and eggs (liver, intestines) collection	Necropsy and PCR	18/498^***^ ^(3.6%)^	Muridae (*A. niloticus* and *M. huberti)*	*S. mansoni,* *S. haematobium*	[40]
9	Kwansa-Bentum et al.,2025	Ghana	Cross-sectional (NHM and human)	Simple random sampling	Wildlife nonhuman primate mammals (Cercopithecidae *n* = 87)	Living animal’s feces	Microscopy (formol-ether concentration)	2/87 (2.3%)	Cercopithecidae (*Cercopithecus mona*)	*S. mansoni*	[39]
10	Larbi et al., 2021	Ghana	Cross-sectional (NHM)	Convenience sampling	Wildlife nonhuman primate mammals: (Cercopithecidae,* n* = 19 and Suidae, *n* = 23)	Living animal’s feces	Microscopy (wet mount, formol ether concentration)	12/42 (28.6%)	Cercopithecidae (*Papio anubis n* = 5) and Suidae (*Phacochoerus aethiopicus* *n* = 7)	*S. mansoni*	[48]
11	Mafuyai et al*.,* 2013	Nigeria	Cross-sectional (NHM)	Convenience sampling	Wildlife nonhuman primate (Cercopithecidea, *n* = 23)	Living animal’s feces	Microscopy (formol-ether concentration)	2/23*** (8.7%)	Cercopithecidea (*P. Anubis*)	*S. mansoni*	[49]
12	Mbaya et al*.*, 2011	Nigeria	Cross-sectional (NHM)	Convenience sampling	Captive and wildlife nonhuman primate: (Hominidae, *n* = 28 and Cercopithecidea,* n* = 80)	Living animal’s feces	Microscopy (flotation and modified Baerman technique	10/108 (9.3%)	Hominidae (*Pan troglodytes*)	*S. mansoni*	[42]
13	McGrew et al., 1989	Senegal	Repetitive cross-sectional (NHM)	Simple random sampling	Wildlife nonhuman primate mammals: (Hominidae, *n* = 70 and Cercopithecidae, *n* = 39)	Living animal’s feces	Microscopy (formol-ether concentration and flotation)	9/109 (8.3%)	Cercopithecidea (*P. papio*)	*S. mansoni*	[50]
14	Nwoha R.I.O and Agu C.V, 2019	Nigeria	Cross-sectional (NHM)	Simple random and convenience sampling	Domestic dogs (Canidae, *n* = 184)	Living animals’ feces collection and anal swab	Microscopy (flotation, direct swab)	3/184 (1.6%)	Canidae (Domestic dogs: *Canis lupus familiaris*)	*S. mansoni*	[51]
15	Omonona *et al*., 2020	Nigeria	Cross-sectional (NHM)	Convenience sampling	Wildlife small mammals Squirrel (Sciuridae, *n* = 33)	Postmortem worms and eggs collection	Microscopy (sedimentation and McMaster slide technique)	1/33 (3.0%)	Sciuridae** (*Funisciurus anerythrus* or *Heliosciurus gambianus*)	*S. intercalatum*	[52]
16	Savassi et al., 2021	Benin	Cross-sectional (NHM and human)	Convenience sampling	Wildlife small mammals (Muridae,* n* = 62)	Postmortem worms and eggs’ (liver and duodenum) collection	Necropsy, microscopy (saline solution), and PCR	6/50*** (12.0%)	Muridae *(M. natalensis* and *Rattus rattus)*	*S. haematobium, S. haematobium* × *S. bovis*	[37]
17	Savassi et al., 2020	Benin	Cross-sectional (NHM and human)	Convenience sampling	Livestock large ruminant mammals (Bovidae*, n* = 48)	Living animals’ feces collection	Microscopy (miracidia hatching test) and PCR	35/48 (72.9%)	Bovidae (Cows: *Bos taurus*)	*S. haematobium* × *S. bovis,*	[38]
18	Savassi A.E.S.B., 2020	Benin	Cross-sectional (NHM, humans, and snails)	NA	Livestock large ruminant mammals and wildlife small mammals (Bovidae, *n* = 157 and Muridae,* n* = 5)	NA	NA	89/162 (54.9%)	Bovidae (cows: *B. taurus*), and Muridae (*M. natalensis*)	*S. haematobium* × *S. bovis*	[36]
19	Taylor et al., 1972	Senegal	Case study (NHM)	Purposive and convenience sampling	Wildlife nonhuman primate mammals: (Cercopithecidae,* n* = 39)	Live animal’s feces and postmortem examination	Necropsy and microscopy (not specified)	1/39 (2.6%)	Cercopithecidae (*Papio* spp.)	*S. haematobium*	[53]
20	Weyher et al., 2006	Nigeria	Cross-sectional (NHM)	Purposive sampling	wildlife nonhuman primate mammals: (Cercopithecidae, *n* = 23)	Living animal’s feces	Microscopy (Formol ether concentration)	1/23 (4.3%)	Cercopithecidae (*P. anubis*)	*S. mansoni*	[54]

*Specimen counts (animal droppings) exceed individual animal counts because of passive environmental collection methods. ** NHM host species could not be determined for some positive cases. ***Discrepancies between surveyed and screened animal numbers reflect potential resampling of individuals;* NA* indicates unavailable data, *NHM* Nonhuman mammal

Discrepancies between specimen counts and individual animal numbers reflect study-specific methodologies: (1) some studies analysed multiple samples per animal (e.g., repeated faecal collections), whereas (2) environmental sampling of communal droppings prevented exact host enumeration. These methodological variations were preserved in our synthesis to maintain the original reporting formats of the study authors, although they preclude direct per-animal prevalence comparisons across studies. All extracted data explicitly note whether samples or individual animals were counted

### Biological sampling and diagnostic approaches

Studies have employed multiple biological samples: living animal feces (rectal or freely passed), anal swabs, and postnecropsy organ samples (liver, intestines, duodenum, kidneys, lungs, and spleen) potentially containing adult worms or eggs (Table 2). All the articles specified diagnostic methods, which included (1) microscopy (10 studies; using Kato-Katz, wet mounts, saline swabs, miracidia hatching, or stool concentration via flotation/Mini-FLOTAC/formalin-ether); (2) necropsy for direct worm/egg detection; and (3) polymerase chain reaction (PCR) targeting cytochrome c oxidase subunit 1 (COX1) and internal transcribed spacer (ITS) regions for species confirmation/phylogenetics. Combined approaches were common: microscopy + necropsy (3 studies), microscopy + PCR (1), all three methods (2), and necropsy + PCR (3). Light microscopy remains the predominant standalone technique (50% of studies).

### Epidemiological patterns of zoonotic schistosomiasis transmission

**Fig. 3:**
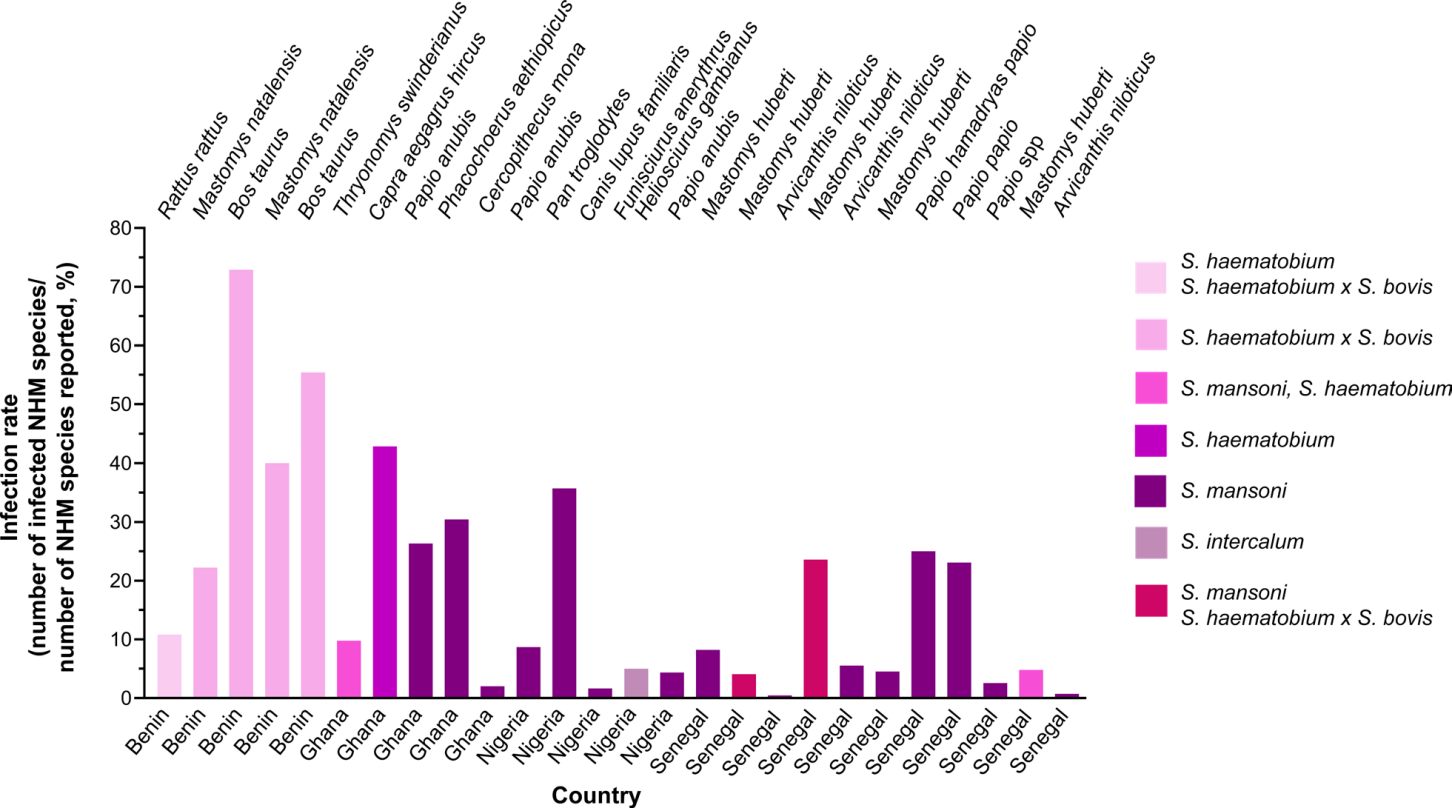
Distribution of human-infective schistosomes in nonhuman mammalian reservoirs across western Africa (1972–2025). *NHM* Nonhuman mammal. This distribution is generated via GraphPad Prism 10.6.1 (Dotmatics, Boston, USA)

Among the 4,849 surveyed NHMs, 378 confirmed infections had an 8% prevalence (95% *CI*: 7–9%), with striking geographic variation: Benin had the highest infection rate (50%), followed by Ghana (9%), whereas Nigeria and Senegal presented a 5% prevalence each (Fig. 2B). Sixteen NHM species (*Arvicanthis niloticus*, *Bos taurus*, *Canis lupus familiaris*, *Capra aegagrus hircus*, *Cercopithecus mona*, *Mastomys huberti*, *M. natalensis*, *Thryonomys swinderianus*, *Phacochoerus aethiopicus*, *Pan troglodytes*, *Papio anubis*, *P. hamadryas papio*, *Heliosciurus gambianus*, *Funisciurus anerythrus*, *Ovis aries,* and *Rattus rattus*) harbored HISs. *Bos taurus* represented the most frequently infected reservoir (60%), followed by *C. aegagrus hircus* (43%) and *P. troglodytes* (36%) (Fig. 3). *S. mansoni* predominated approximately 47% of the cases and infected nine NHM species across Ghana, Nigeria, and Senegal. *S. haematobium* × *S. bovis* hybrids account for 33% of infections, primarily in Benin and Senegal, and circulate among rodents (*R. rattus* and *Mastomys* spp.) and cattle (*B. taurus*). Classical *S. haematobium* infections have emerged in Benin and Ghana (infecting rodents: *R. rattus*, *T. swinderianus*; and goats: *C. eagragus hircus*), whereas *S. intercalatum* occurs exclusively in Nigerian squirrels (*F. anerythrus*, *H. gambianus*). One study [51] reported only proportional data and was excluded from absolute calculations.

While *Bos taurus* presented the highest observed infection rate (60%), its ability to serve as a reservoir for human transmission remains unresolved. Current evidence from Senegal suggests that cattle-derived hybrids can infect humans [32], but their relative contribution to anthroponotic transmission is unquantified. Critical knowledge gaps persist regarding (i) egg viability/shedding rates in cattle versus human hosts and (ii) the efficiency of cattle-snail-human transmission cycles under field conditions. These uncertainties underscore the need for paired human‒livestock molecular epidemiological studies to quantify spillover risk in high-prevalence zones.

## Discussion

While previous studies have examined zoonotic schistosomiasis in Africa [29, 32], this review provides the first comprehensive synthesis focused specifically on West Africa's unique transmission ecology, where the convergence of endemic human schistosomes, livestock reservoirs, and emerging hybrid strains presents distinct elimination challenges. Our analysis of 20 studies revealed two critical, region-specific findings: (i) the disproportionate role of livestock (particularly *Bos taurus*) as reservoirs for *S. haematobium* × *S. bovis* hybrids and (ii) the wide distribution of *S. mansoni* across rodent and primate hosts—patterns that differ markedly from eastern/southern African contexts. By systematically mapping these West African-specific transmission dynamics, we provide an evidence base for tailoring WHO-recommended One Health strategies to the region's ecological and socioeconomic realities.

Current schistosomiasis control programs in West Africa remain heavily reliant on school-based preventive chemotherapy [16, 17], which has successfully reduced the prevalence of schistosomiasis [55] but faces limitations in achieving elimination targets. While this approach has decreased heavy infection intensities in school-aged children, the ambitious goals of reducing the WHO prevalence to < 1% and interrupting transmission by 2030 [19] require addressing zoonotic reservoirs that may sustain parasite circulation. Our review reveals significant gaps in understanding and addressing NHM reservoirs in western Africa, which contrasts sharply with the well-documented zoonotic transmission in Asia, where over 40 NHM species maintain *S. japonicum* [56].

Diagnostic approaches in the reviewed studies relied heavily on traditional methods [57], with microscopy alone used in 50% of cases. While necropsy with microscopy/PCR has proven effective for small mammals (rodents and shrews) [58], we identified opportunities to increase sensitivity through the adoption of validated molecular techniques such as quantitative polymerase chain reaction (qPCR) and loop-mediated isothermal amplification (LAMP), which have shown superior performance in human studies [57, 59]. The absence of antigen detection methods (e.g., CCA) and advanced molecular tools [60] in NHM studies represents a key technological gap that may lead to underestimation of the true prevalence, particularly for low-intensity infections [57].

Hybrids of *S. haematobium* × *S. bovis* were reported in Benin and Senegal. Sexual dimorphism is a remarkable trait in schistosomes, in contrast to the mostly hermaphroditic nature of most other trematodes [25]. Schistosome species exhibit geographical and host range overlap, facilitating interspecies pairing of male and female schistosomes [25]. The evolutionary distance between two species may result in parthenogenesis or hybridization, with some combinations exhibiting greater viability than others do [25]. The two closely related species (belonging to the *S. haematobium* group), *S. haematobium* and *S. bovis,* produce viable offspring, as reported in Senegal [61]. Under experimental conditions, *S. haematobium* × *S. bovis* produce viable offspring up to F3 [25]. Hybrids of *S. haematobium* × *S. bovis* have been found infecting humans, livestock, wildlife, and the snail intermediate host *Bulinus truncatus *[25, 61–64]*.* The high prevalence of *S. haematobium* × *S. bovis* hybrids in Benin and Senegal likely reflects synergistic ecological and genomic drivers: (i) intense livestock-water contact patterns in pastoral and lacustrine communities create frequent transmission interfaces [32]; (ii) genomic compatibility between bovine and human *Schistosoma* species facilitates viable hybrid formation, as demonstrated by mitochondrial/nuclear interactions in Mali [35]; and (iii) agricultural intensification may select for hybrid vigor, with preliminary evidence suggesting that hybrids exhibit broader intermediate host specificity [65]. These mechanisms—operating most intensely in western Africa's mixed farming systems—may explain the region's disproportionate hybridization rates compared with eastern/southern Africa. Molecular surveillance tracking COX1 and ITS markers should be prioritized to monitor potential hybrid expansion into new ecological niches. The frequent detection of *S. haematobium* × *S. bovis* hybrids in western Africa contrasts with patterns in eastern Africa and raises concerns about transmission, epidemiology, and pathogenicity [66–68]. Historical evidence suggests that these hybrids may be spreading eastwards, with documented human cases as far as France [65].

Beyond their documented prevalence, the profound threat posed by *S. haematobium* × *S. bovis* hybrids lies in their unique biological properties—their genesis, reproductive strategy, and epidemiological trajectory—which collectively challenge the foundations of current control paradigms [61].

First—Initial genesis and host: The genesis of these hybrids is a two-host process, initiated when a mammalian host (e.g., cattle or humans) is coinfected with both *S. haematobium* and *S. bovis*. Within this definitive host, reciprocal cross-fertilization between the two species can occur, resulting in the production of hybrid eggs. The viability of these hybrids is ultimately determined in the snail intermediate host, which must be susceptible to infection by the hybrid miracidia for the life cycle to perpetuate.

Second—Reproductive potential: In contrast to the sterility observed in the hybrid *Fasciola*, many schistosome hybrids demonstrate a formidable reproductive capacity. They leverage a dual strategy: facultative asexual clonal amplification within the snail host, which ensures massive cercarial output, and the ability to backcross with parental species in subsequent generations. This reproductive plasticity not only guarantees their persistence but also actively facilitates introgression, allowing adaptive traits to sweep through parasite populations.

Third—Vector preference and fitness: Emerging evidence points to a critical "hybrid vigour" in transmission dynamics. Some *S. haematobium* × *S. bovis* hybrids exhibit broader intermediate host specificity, potentially infecting a wider range of *Bulinus* snail species than either parent does. This expanded ecological niche reduces key transmission bottlenecks and may significantly increase geographic spread and resilience in changing environments.

Fourth—Pathogenicity and spreading trends: The implications for human health are profound, as these hybrids may exhibit altered virulence and clinical manifestations. Molecular surveillance has already documented their troubling expansion from initial hotspots in Senegal and Mali, which spread both westwards and eastwards. This trend signals an evolving epidemiological landscape where hybrid-derived morbidity could become increasingly common, undermining progress towards elimination.

## Limitations

This study provides the first comprehensive synthesis of NHM reservoirs in western Africa; however, several limitations constrain the generalizability of the findings. While it synthesizes critical evidence on zoonotic schistosomiasis in western Africa, the geographic scope is limited to four countries (Benin, Ghana, Nigeria, Senegal), which may not fully represent regional transmission dynamics. High-burden countries such as Mali and Niger—where hybrid *S. haematobium* × *S. bovis* strains and livestock reservoirs are increasingly reported [32, 35]—are underrepresented due to a lack of eligible studies. This gap likely stems from disparities in research capacity, surveillance infrastructure, or publication bias toward anglophone regions. Consequently, our prevalence estimates and reservoir host profiles may not extend to ecologically distinct zones (e.g., Sahelian pastoral systems or urban-periurban interfaces). Future surveillance should prioritize data-poor countries to refine elimination strategies across western Africa’s diverse transmission landscapes.

Methodological inconsistencies across studies—particularly the predominance of convenience sampling (65% of studies) and reliance on low-sensitivity diagnostics (microscopy alone in 50% of studies)—likely underestimate true prevalence and obscure transmission dynamics. The absence of standardized reporting (e.g., only 22% of studies quantified parasite loads) limits the assessment of NHMs’ actual contribution to human transmission. Furthermore, the cross-sectional design of all included studies precludes evaluation of temporal trends or seasonal variation in zoonotic spillover. The predominance of microscopy-based diagnostics (50% of the included studies) likely underestimates the true prevalence of HIS in NHM reservoirs, particularly for low-intensity infections and hybrid strains. While microscopy remains accessible in resource-limited settings, its sensitivity limitations are well documented [59, 60]. Future studies should prioritize molecular tools such as qPCR and LAMP, which offer superior sensitivity and specificity for detecting schistosome DNA in both fecal and tissue samples [57]. These methods are particularly critical for differentiating hybrid strains (e.g., *S. haematobium* × *S. bovis*) and quantifying zoonotic transmission risks. Integrating molecular diagnostics into routine NHM surveillance will not only improve accuracy but also align with the WHO's One Health framework by enabling harmonized data collection across the human, animal, and environmental sectors. Together, these gaps underscore the need for harmonized, multicountry surveillance integrating molecular diagnostics (qPCR/LAMP) and One Health frameworks to robustly quantify the role of NHMs in schistosomiasis elimination efforts.

## Recommendations

While livestock treatment in hybrid-endemic areas represents a potentially impactful intervention, its implementation faces three key challenges: (i) economic constraints, as mass praziquantel administration to cattle would require substantial budget reallocations in already underfunded NTD programs; (ii) stakeholder engagement complexities, needing coordination between veterinary and public health sectors with competing priorities; and (iii) ecological concerns regarding drug resistance development in both human and animal schistosome populations. Pilot programs should therefore first evaluate cost-effective targeted approaches—such as treating only cattle near human water contact points—while establishing monitoring systems for treatment efficacy and potential resistance emergence.

The absence of eligible studies from 2022–2023 may reflect multiple factors: (i) publication lags inherent in peer-reviewed research, particularly for field-based zoonotic studies requiring extensive laboratory confirmation; (ii) disruptions to field research activities during the COVID-19 pandemic, which disproportionately affected NTD programs in West Africa [55]; and (iii) potential delays in indexing African-published studies in international databases. While this gap limits our ability to assess very recent trends, the consistent reporting of hybrid strains and NHM reservoirs both before and after this period suggests that these findings remain epidemiologically relevant. Future systematic reviews would benefit from proactive inclusion of preprint repositories and regional database searches to capture emerging evidence.

The persistent zoonotic transmission cycles documented here disproportionately affect impoverished communities where (i) subsistence farming necessitates high-risk livestock-water contact, (ii) limited sanitation infrastructure increases environmental contamination, and (iii) healthcare access barriers delay human case detection and treatment. This creates a poverty trap where marginalized populations face compounded risks from both direct schistosome exposure and livestock-associated hybrid strains. Our findings underscore that sustainable elimination will require interventions addressing these socioeconomic determinants alongside biomedical strategies—particularly through integrated human-animal health programmes targeting smallholder farming communities.

## Conclusion

This study highlights the necessity of integrated strategies to eliminate schistosomiasis in western Africa, moving beyond human preventive chemotherapy and addressing zoonotic reservoirs. This suggests implementing coordinated human-NHM-snail monitoring, developing targeted interventions, validating diagnostic advancements, and establishing a One Health platform to coordinate surveillance and intervention efforts.

## Data Availability

The datasets related to the current work are available from the corresponding author upon reasonable request.

## Abbreviations

CCA: Circulating cathodic antigen
COX1: Cytochrome oxidase subunit 1
HIS: Human-infective schistosome
ITS: Internal transcribed spacer
LAMP: Loop-mediated isothermal amplification
MDA: Mass drug administration
NHM: Nonhuman mammal
NTD: Neglected tropical disease
PCR: Polymerase chain reaction
PRISMA: Preferred reporting items for systematic reviews and meta-analyses
qPCR: Quantitative polymerase chain reaction
SSA: Sub-saharan Africa
WHO: World health organization DALY: disability-adjusted life year
DALY: Disability-adjusted life year
